# Heterosubtypic immunity increases infectious dose required to infect Mallard ducks with Influenza A virus

**DOI:** 10.1371/journal.pone.0196394

**Published:** 2018-04-26

**Authors:** Karen M. Segovia, Monique S. França, Christina L. Leyson, Darrell R. Kapczynski, Klaudia Chrzastek, Charlie S. Bahnson, David E. Stallknecht

**Affiliations:** 1 Poultry Diagnostic and Research Center, The University of Georgia, Athens, Georgia, United States of America; 2 Southeast Poultry Research Laboratory, Agricultural Research Service, U.S. Department of Agriculture, Athens, Georgia, United States of America; 3 Southeastern Cooperative Wildlife Disease Study, Department of Population Health, College of Veterinary Medicine, University of Georgia, Athens, Georgia, United States of America; Linnaeus University, SWEDEN

## Abstract

Previous field and experimental studies have demonstrated that heterosubtypic immunity (HSI) is a potential driver of Influenza A virus (IAV) prevalence and subtype diversity in mallards. Prior infection with IAV can reduce viral shedding during subsequent reinfection with IAV that have genetically related hemagglutinins (HA). In this experiment, we evaluated the effect of HSI conferred by an H3N8 IAV infection against increasing challenge doses of closely (H4N6) and distantly (H6N2) related IAV subtypes in mallards. Two groups of thirty 1-month-old mallards each, were inoculated with 10^5.9^ 50% embryo infectious doses (EID_50_) of an H3N8 virus or a mock-inoculum. One month later, groups of five birds each were challenged with increasing doses of H4N6 or H6N2 virus; age-matched, single infection control ducks were included for all challenges. Results demonstrate that naïve birds were infected after inoculation with 10^3^ and 10^4^ EID_50_ doses of the H4N6 or H6N2 virus, but not with 10^2^ EID_50_ doses of either IAV. In contrast, with birds previously infected with H3N8 IAV, only one duck challenged with 10^4^ EID_50_ of H4N6 IAV was shedding viral RNA at 2 days post-inoculation, and with H6N2 IAV, only birds challenged with the 10^4^ EID_50_ dose were positive to virus isolation. Viral shedding in ducks infected with H6N2 IAV was reduced on days 2 and 3 post-inoculation compared to control birds. To explain the differences in the dose necessary to produce infection among H3-primed ducks challenged with H4N6 or H6N2 IAV, we mapped the amino acid sequence changes between H3 and H4 or H6 HA on predicted three-dimensional structures. Most of the sequence differences occurred between H3 and H6 at antigenic sites A, B, and D of the HA1 region. These findings demonstrate that the infectious dose necessary to infect mallards with IAV can increase as a result of HSI and that this effect is most pronounced when the HA of the viruses are genetically related.

## Introduction

Wild birds are the natural reservoir for all the 16 subtypes of hemagglutinin (H1 to H16) and nine subtypes of neuraminidase (N1 to N9) of Influenza A viruses (IAV) [1–3]. Wild ducks, especially mallards (*Anas platyrhynchos*) are the primary reservoir for most subtypes of IAV [3–8]. Several factors influence the dynamics of IAV in waterfowl such as seasonality, spatial dynamics, and host density [2, 6, 9–11]; however, the drivers for subtype diversity in these populations are unknown [12, 13]. It has been suggested that population immunity related to homo- and heterosubtypic immunity could provide a mechanism for seasonal shifts in subtype predominance and observed variations in subtype prevalence within a given season [14, 15]. Effects related to homo- and heterosubtypic immunity have been reported from experimental IAV infections in mallards [16–20]. Previously, we demonstrated that cross-protective immunity between IAV subtypes results in reduced virus shedding in mallards that is positively associated with the phylogenetic relatedness of the hemagglutinin (HA) of the challenge viruses [21, 22]. It is also possible that previous IAV infections could affect viral transmission within waterfowl populations by increasing the dose necessary to produce subsequent IAV infections. To address this question, dose-response experiments were conducted in one-month-old mallards which were infected with a single dose of H3N8 IAV and subsequently infected one month later with H4N6 or H6N2 IAV. Also, the HA amino acid sequence changes between H3, H4, and H6 subtypes were compared by predicted three-dimensional structures. The purpose of this study was to determine if HSI induced by one subtype of IAV would increase the infectious dose required to infect mallards in subsequent challenges with a different subtype and to determine if this potential effect correlated with the phylogenetic relatedness of the HA of the viruses.

## Materials and methods

### Animals and husbandry

Sixty one-day-old mallards were obtained from a commercial waterfowl supplier (McMurray Hatchery, Webster City, IA, USA). All work was done in accordance with guidelines of the Institutional Animal Care and Use Committee (IACUC) of The University of Georgia under an approved animal use protocol (AUP# A2015 12-002-Y1-A0). All experimental and laboratory work was conducted in biosafety level 2 (ABSL2) facility. Ducks that were relocated to high-efficiency particulate (HEPA) filter isolators were acclimatized for a week before secondary virus inoculations. Animals were provided with food and water ad libitum and monitored twice a day throughout the study. Once the animal experiment was completed, surviving ducks were humanely euthanized by carbon dioxide followed by cervical dislocation.

### Viruses

All three IAV isolates used in this study were obtained from ducks during wild bird surveillance studies in Minnesota, USA: A/mallard/MN/Sg-000169/2007(H3N8), A/Mallard/MN/AI11-4979/2011(H4N6), and A/Mallard/MN/AI11-4982/2011(H6N2). The viruses had undergone two passages in 9- to 10-day-old specific-pathogen-free (SPF) embryonated chicken eggs (ECE) before their use in this experiment. Virus stocks stored at -80°C were thawed and diluted to obtain a 10^6^ 50% embryo infectious dose (EID_50_) per 0.1 ml of the H3N8 virus and 10^2^, 10^3^ and 10^4^ EID_50_/0.1 ml of the H4N6 and H6N2 viruses. Back titrations of the inoculum were performed in SPF ECE on the inoculation day, and the EID_50_ of H3N8 (10^5.9^), H4N6 (10^1.8^, 10^3^, and 10^4.1^ EID_50_/0.1ml), and H6N2 (10^1.9^, 10^3^, and 10^3.9^ EID_50_/0.1ml) viruses were calculated by the Reed and Muench method [23]. The 50% bird infectious doses (BID_50_) were calculated by the same method. A mock-inoculum consisting of viral transport media was used as previously described [21].

### Viral inoculations

Two groups of thirty 1-month-old ducks were inoculated with either 10^5.9^ EID_50_ of an H3N8 IAV or a mock-inoculum in a total volume of 0.1 ml via the choanal cleft. Groups of five birds containing H3N8-primed or mock-inoculated ducks were challenged with 10^1.8^, 10^3^, and 10^4.1^ EID_50_ of the H4N6 or 10^1.9^, 10^3^, and 10^3.9^ EID_50_ of the H6N2 IAV at 2 months of age.

### Sample collection

Oropharyngeal (OP) and cloacal (CL) swabs were collected in viral transport media before each inoculation to confirm the absence of IAV shedding. OP and CL swabs were collected at 3 and 28 days post-inoculation (DPI) with H3N8 IAV to confirm infection and absence of viral shedding, respectively. Swab samplings after challenge with H4N6 or H6N2 were done at 0, 2 to 5, 7, and 14 DPI and samples were stored at -80°C before processing. Individual blood samples were obtained from the jugular vein at 0 and 14 DPI with each virus and stored at -20°C before serological testing.

### Virus isolation and RT-PCR

Virus isolation and real-time reverse-transcriptase polymerase chain reaction (rRT-PCR) were performed after thawing the samples. Details of the methodology for virus isolation (VI), RNA extraction, and rRT-PCR have been previously described [24, 25]. A sample was considered positive to virus isolation if the alantoic fluid hemagglutinated 0.5% chicken red blood cells or the Ct value was ≤ 40.

### Serology

All serum samples were analyzed using the AI MultiS-Screen Ab ELISA kit (cELISA, IDEXX, Westbrook, ME, USA) according to manufacturer's recommendations. Samples were also tested by microneutralization (MN) assays as previously described [26]; the viruses A/mallard/MN/AI10-2593/2010 (H3N8), A/mallard/MN/AI10-3208/2010 (H4N6), and A/mallard/MN/SG-01048/2008 (H6N1) were used as antigens.

### Statistical analysis

Comparison of Ct values between control and H3N8-primed groups over time to assess differences in viral RNA loads was done using linear mixed models with repeated measurements. Samples with undetermined Ct values were assigned values of 45 for statistical analyses. The Bonferroni correction was used to limit the type I error rate to 5%. Differences in the MN antibody titers among experimental groups were done by using non-parametric tests (Kruskal-Wallis or Mann-Whitney U). STATA software version 14.0 (StataCorp LP, College Station, TX) was used for statistical analyses, and the graphs were generated with GraphPad Prism software version 6.0 (GraphPad Software Inc., San Diego, CA, USA).

### Sequence alignment

Sequences of the HA and NA of the viruses used for the challenges were downloaded from the GenBank database (accession numbers CY035285, CY042134, MF664431, MF664433, KX814374, KX814375). Multiple sequence alignment of the HA and NA were constructed with MAFFT, and an amino acid distance matrix was obtained by using Geneious version 8.1.9 (Biomatters Ltd; Auckland, New Zealand).

### Prediction of hemagglutinin molecular structure and antibody epitopes

Amino acid sequences obtained from GenBank were subjected to homology modeling using I-TASSER [27]. The signal peptide (amino acid positions 1–18) were removed from the sequences submitted to the I-TASSER server. PyMoL was then used to visualize molecular structures [28]. Continuous antibody epitopes were predicted using the Kolaskar and Tongaonkar method [29], as made available in the Geneious software 8.1.9 (Biomatters Ltd; Auckland, New Zealand). Additional image processing was also performed using Adobe Photoshop CS6 (Adobe Systems Inc.).

## Results

### Viral shedding and BID_50_

Ducks did not present overt clinical signs during the duration of the experiment. All thirty 1-month-old mallards inoculated with 10^5.9^ EID_50_ of H3N8 IAV became infected, as all birds were detected positive at 3 DPI by VI and RT-PCR in OP and CL swabs. Viral shedding ended by 28 DPI and control birds remained negative for IAV during this part of the experiment.

**Fig 1:**
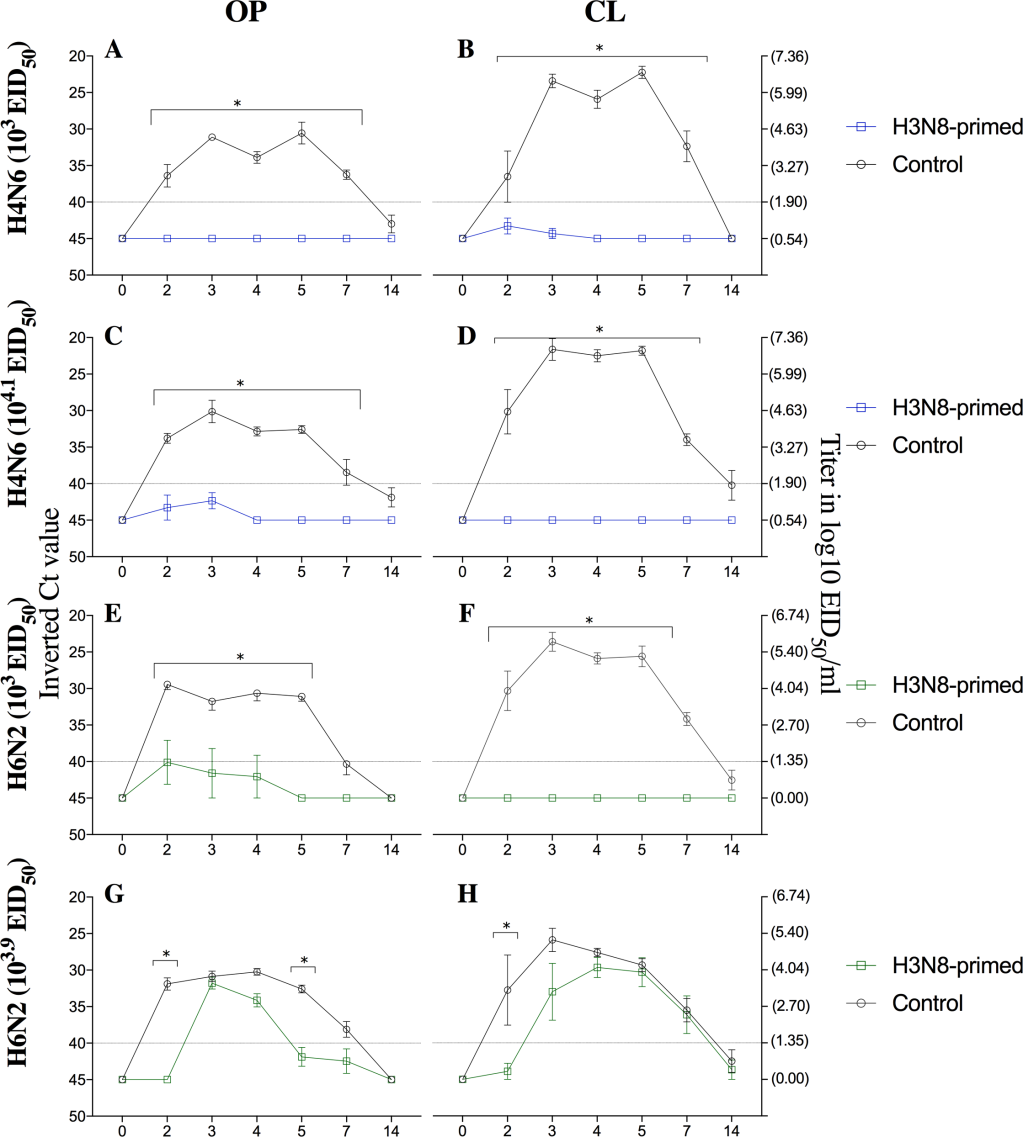
Ct values after inoculation of H3N8-primed and control mallards with increasing doses of H4N6 and H6N2 LPAIV. Graphs compare the duration of viral RNA shedding (Ct values) detected at 2–5, 7, and 14 DPI from OP (left) and CL (right) swabs after A, B) H4N6 (10^3^EID_50_); C,D) H4N6(10^4.1^EID_50_); E,F) H6N2(10^3^EID_50_) and G,H) H6N2(10^3.9^EID_50_) inoculation. * denotes significant differences (P < 0.05) between H3N8-primed and control birds.

Control and H3N8-primed ducks were not susceptible to infection with 10^1.8^ EID_50_ of the H4N6 or 10^1.9^ EID_50_ of the H6N2 IAV at two months of age, as none of them had detectable virus in OP or CL swabs as determined by VI and rRT-PCR (Table 1). Also, all ducks inoculated with H3N8 IAV, followed by 10^3^ and 10^4.1^ EID_50_ doses of H4N6 virus were negative for IAV by VI (Table 1). Only one OP swab from the group inoculated with the 10^4.1^ EID_50_ dose was positive by rRT-PCR at 2 DPI. Significant differences in Ct values of H4N6 IAV among H3N8-primed and control groups were observed from 2 to 7 DPI for OP and CL swabs in both groups (P<0.05) (Fig 1).

**Table 1:** Viral shedding in H3N8-primed and control birds with increasing doses of two LPAIV. The table shows the proportion of samples positive for H4N6 or H6N2 IAV by VI in OP and CL swabs on 0, 2–5, 7 and 14 DPI after inoculation with increasing doses (EID_50_) of two LPAIV.

Challenge groups	0 DPI		2 DPI		3 DPI		4 DPI		5 DPI		7 DPI		14 DPI	
	OP	CL	OP	CL	OP	CL	OP	CL	OP	CL	OP	CL	OP	CL
H3N8X**H4N6(10**^**1.8**^**)**	0/5	0/5	0/5	0/5	0/5	0/5	0/5	0/5	0/5	0/5	0/5	0/5	0/5	0/5
**H4N6(10**^**1.8**^**)**	0/5	0/5	0/5	0/5	0/5	0/5	0/5	0/5	0/5	0/5	0/5	0/5	0/5	0/5
H3N8X**H4N6(10**^**3**^**)**	0/5	0/5	0/5	0/5	0/5	0/5	0/5	0/5	0/5	0/5	0/5	0/5	0/5	0/5
**H4N6(10**^**3**^**)**	0/5	0/5	3/5	2/5	5/5	5/5	5/5	5/5	4/5	5/5	1/5	1/5	0/5	0/5
H3N8X**H4N6(10**^**4.1**^**)**	0/5	0/5	0/5	0/5	0/5	0/5	0/5	0/5	0/5	0/5	0/5	0/5	0/5	0/5
**H4N6(10**^**4.1**^**)**	0/5	0/5	5/5	5/5	4/5	5/5	5/5	5/5	5/5	5/5	1/5	2/5	0/5	0/5
H3N8X**H6N2(10**^**1.9**^**)**	0/5	0/5	0/5	0/5	0/5	0/5	0/5	0/5	0/5	0/5	0/5	0/5	0/5	0/5
**H6N2(10**^**1.9**^**)**	0/5	0/5	0/5	0/5	0/5	0/5	0/5	0/5	0/5	0/5	0/5	0/5	0/5	0/5
H3N8X**H6N2(10**^**3**^**)**	0/5	0/5	0/5	0/5	0/5	0/5	0/5	0/5	0/5	0/5	0/5	0/5	0/5	0/5
**H6N2(10**^**3**^**)**	0/5	0/5	4/5	5/5	1/5	5/5	5/5	5/5	3/5	5/5	0/5	1/5	0/5	0/5
H3N8X**H6N2(10**^**3.9**^**)**	0/5	0/5	0/5	2/5	2/5	2/5	1/5	5/5	1/5	4/5	1/5	2/5	0/5	0/5
**H6N2(10**^**3.9**^**)**	0/5	0/5	4/5	5/5	2/5	5/5	5/5	5/5	3/5	5/5	0/5	2/5	0/5	0/5

Ducks primed with H3N8 IAV did not shed viable virus in OP or CL swabs after inoculation with 10^3^ EID_50_ of H6N2 virus; however, two out of five birds shed viral RNA and had positive Ct values in OP swabs at 2 DPI. Viral excretion was delayed by 2 to 3 days in three birds inoculated with the 10^3.9^ EID_50_ as detected by VI in OP and CL swabs. Significant differences in Ct values between control and H3N8-primed groups in OP and CL swabs after inoculation with the H6N2 IAV were observed from 2 to 5 DPI in the 10^3^ EID_50_ dose group, and at 2 DPI in the 10^3.9^ EID_50_ group (P<0.05) (Fig 1).

The calculated BID_50_ for both H4N6 and H6N2 viruses in naïve two-month-old mallards was 10^3^ EID_50_. For mallards previously infected with H3N8, the BID_50_ increased to 10^3.9^ for H6N2 virus and was greater than 10^4.1^ EID_50_ for the H4N6 virus.

### Serology

All ducks tested negative to IAV before any virus challenge and seroconverted after inoculation with the H3N8 IAV by 14 DPI as determined by cELISA (S1 Table). Consistent with virus isolation results, none of the control birds inoculated with 10^1.8^ EID_50_ of the H4N6 or 10^1.9^ EID_50_ of H6N2 seroconverted by cELISA and MN tests. All control birds inoculated with 10^3^ and approximately 10^4^ EID_50_ of the H4N6 and H6N2 seroconverted by cELISA at 14 DPI (S1 Table).

Neutralizing antibodies against the homologous antigen (H4N6 or H6N2) were detected in control birds challenged with the 10^3^ and approximately 10^4^ EID_50_ doses of the respective virus (Tables 2 and S2). Among the H3N8-primed groups, neutralizing antibodies were only detected in birds challenged with the 10^4^ EID_50_ dose of the H6N2 virus. Heterosubtypic inoculations did not have a boosting effect in the MN antibodies previously induced by the H3N8 virus as differences in the geometric mean titers were not significantly different in samples collected pre- and post- heterosubtypic challenge (P>0.05) (Tables 3 and S3).

**Table 2:** Microneutralization titers after heterosubtypic LPAIV inoculation against H4N6 or H6N2 antigens. The table shows the proportion of positive samples and Log2 microneutralization (MN) titers (media and range) induced against the H4N6 or H6N2 antigens before or after inoculation of control or H3N8-primed ducks with increasing doses of the corresponding virus.

Challenge groups	MN Antigen	0 DPI	Median and range Log_2_ titers	14 DPI	Median and range Log_2_ titers
H3N8X**H4N6(10**^**1.8**^**)**	H4N6	0/5	0	0/5	0
**H4N6(10**^**1.8**^**)**		0/5	0	0/5	0
H3N8X**H4N6(10**^**3**^**)**		0/5	0	0/5	0
**H4N6(10**^**3**^**)**		0/5	0	4/5	6.32 (0–7.32)
H3N8X**H4N6(10**^**4.1**^**)**		0/5	0	0/5	0
**H4N6(10**^**4.1**^**)**		0/5	0	3/5	4.32 (0–5.32)
H3N8X**H6N2(10**^**1.9**^**)**	H6N2	0/5	0	0/5	0
**H6N2(10**^**1.9**^**)**		0/5	0	0/5	0
H3N8X**H6N2(10**^**3**^**)**		0/5	0	0/5	0
**H6N2(10**^**3**^**)**		0/5	0	3/5	4.32 (0–6.32)
H3N8X**H6N2(10**^**3.9**^**)**		0/5	0	3/5	4.32 (0–7.32)
**H6N2(10**^**3.9**^**)**		0/5	0	5/5	5.32 (5.32–7.32)

**Table 3:** Microneutralization titers after heterosubtypic LPAIV inoculation against H3N8 antigen. The table shows the proportion of positive samples and Log_2_ microneutralization (MN) titers (media and range) induced against H3N8 virus before and after inoculation of control or H3N8-primed mallards with increasing doses of H4N6 or H6N2 virus.

Challenge groups	MN Antigen	0 DPI	Median and range Log_2_ titers	14 DPI	Median and range Log_2_ titers
H3N8X**H4N6(10**^**1.8**^**)**	H3N8	5/5	4.32	3/5	4.32 (0–5.32)
**H4N6(10**^**1.8**^**)**		0/5	0	0/5	0
H3N8X**H4N6(10**^**3**^**)**		4/5	4.32 (0–5.32)	3/5	4.32 (0–4.32)
**H4N6(10**^**3**^**)**		0/5	0	0/5	0
H3N8X**H4N6(10**^**4.1**^**)**		3/5	4.32 (0–5.32)	3/5	4.32 (0–6.32)
**H4N6(10**^**4.1**^**)**		0/5	0	0/5	0
H3N8X**H6N2(10**^**1.9**^**)**		2/5	0 (0–4.32)	3/5	4.32 (0–4.32)
**H6N2(10**^**1.9**^**)**		0/5	0	0/5	0
H3N8X**H6N2(10**^**3**^**)**		3/5	4.32 (0–7.32)	4/5	4.32 (0–5.32)
**H6N2(10**^**3**^**)**		0/5	0	0/5	0
H3N8X**H6N2(10**^**3.9**^**)**		4/5	4.32 (0–6.32)	3/5	6.32 (0–6.32)
**H6N2(10**^**3.9**^**)**		0/5	0	0/5	0

### Sequence alignment, predicted hemagglutinin molecular structure, and antibody epitopes

The HA amino acid sequence of the H3N8 virus was 68.3% similar to the H4N6 virus and 41.9% similar to the H6N2 virus, while the similarity among the NA sequences of these viruses were 43.5% and 45.3%, respectively. In addition, most of the differences between subtypes were found in the HA1 region of the IAV with 57.1% similarity between the H3 and H4 HA and 37.5% between H3 and H6.

**Fig 2:**
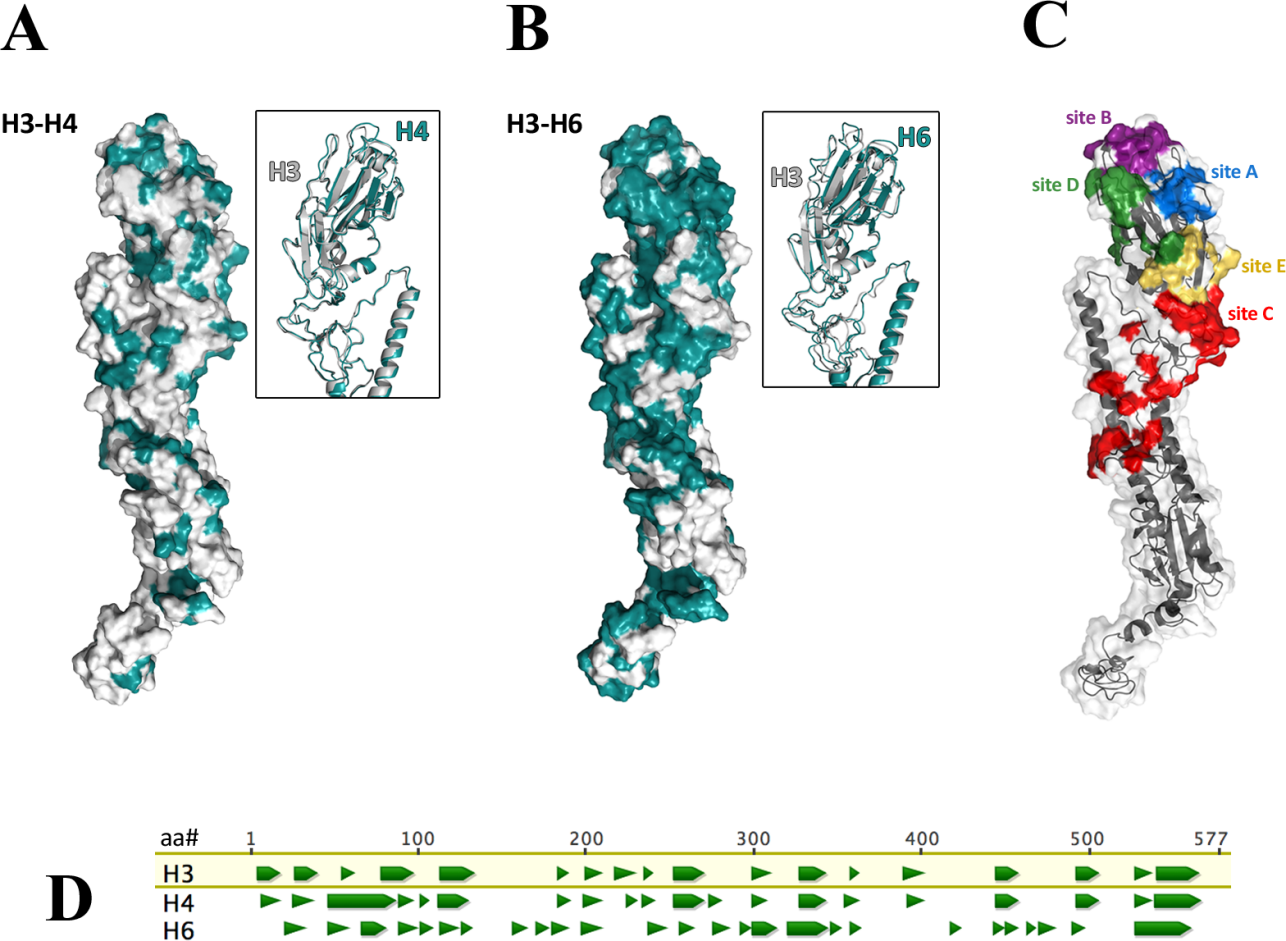
Predicted three-dimensional structures of the hemagglutinin protein (HA0) were obtained using I-TASSER and predicted continuous epitopes were obtained through the Kolaskar and Tongaonkar method. Differences in the amino acid sequence between H3 and H4 (A), and between H3 and H6 (B) are highlighted in teal. Known antigenic sites of H3 are mapped on the predicted structure of H3 hemagglutinin (C). Continuous antibody epitopes were predicted using the Kolaskar and Tongaonkar method (D). H3, H4, and H6 amino acid sequences were aligned and antigenicity at each amino acid position was calculated using the Kolaskar and Tongaonkar method. Amino acid positions that have a positive antigenicity index, i.e. amino acid positions that are predicted to be continuous antibody epitopes, are represented as green triangles.

We used I-TASSER to generate a predicted molecular model for the hemagglutinin (HA0) of the three viruses utilized in this study (Fig 2). On the predicted H3 model, we marked the amino acid positions that contained a residue that was not identical to that found in either the H4 or H6 HA sequences. Additionally, we mapped antigenic sites that were previously described for avian influenza viruses bearing H3 [30]. As expected, we observed that there are more sequence differences among H3 and H6 HA as compared to H3 and H4 (Fig 2A and 2B). Many of these sequence changes are found in the HA1 region, particularly in antigenic sites A, B, and D (Fig 2A–2C). Notably, these antigenic sites are found at the globular head domain where the receptor binding and vestigial esterase regions are found [31]. Comparison of the continuous antibody epitope prediction using the Kolaskar and Tongaonkar method (Fig 2D) indicated that predicted antigenic regions are more similar between H3 and H4 compared to that between H3 and H6.

## Discussion

In previous studies, we demonstrated that infection of mallards with an IAV could result in reduced viral shedding after subsequent heterosubtypic IAV inoculations. In addition, the magnitude of this effect during repeated challenges increased with the genetic relatedness of the HA of the viruses [21, 22]. Here we expand that study to determine whether the same effect was observed with infectious dose. Inoculation of mallards with the H3N8 virus increased the minimum dose required to produce subsequent infection with a closely (H4N6) or distantly related (H6N2) IAV. In addition, the extent of this effect was enhanced if the HA of the viruses used for the challenges were closely related. Indeed, examination of the amino acid sequence changes among the HAs of the H3N8 and H4N6 or H6N2 viruses showed more sequence differences between the HA of the H3N8 and H6N2 viruses. Additionally, most of these differences were found at antigenic regions A, B, and D close to the globular head of the HA.

Comparison of the predicted antibody epitopes in the three hemagglutinin genes examined showed that H3 and H4 hemagglutinins share more regions identified as potential antibody epitopes as compared to H3 and H6 hemagglutinins.

The probability of isolation of IAV from young wild ducks is higher than adults regardless of the time of the year the samples were collected [32]. Also, seasonal variations in both IAV prevalence and subtype diversity have been observed in ducks in North America [3, 33]. In these populations, the H3 and H4 IAV subtypes predominate at the end of the summer and beginning of the fall coinciding with the annual peak of IAV prevalence in ducks [33–35]. The observed subtype prevalence shifts annually to H7 and H10 during the spring migration [14]. These variations may relate to the combined effects of partial cross-protection induced and waning immunity over time [15, 33]. Our results and previous studies [21, 22] are consistent with these field observations as both a reduction in viral shedding and an increase in the dose required to produce subsequent IAV infection were observed. These effects would potentially reduce the transmission of IAV within duck populations and reduce the probability of detecting the virus during subsequent infections in nature.

In our study, none of the ducks receiving the lowest dose of the H4N6 or H6N2 virus was infected. Similar results were reported for Pekin ducks that were inoculated via the intranasal route with two H4N8 IAV strains, where higher BID_50_ (10^3.1^–10^3.3^ EID_50_) were required for infection [36]. In contrast, lower doses (10^1.9^ EID_50_) were required when H5N1 LPAIV were used as inoculum [36]. Our findings suggest that there is a viral load threshold required to infect mallards with IAV that can be increased if birds were previously infected with other IAV. This effect might influence the probability of transmission of IAV when birds are experimentally or naturally exposed to low concentrations in IAV contaminated environments. However, it should be noted that the IAV utilized here had undergone two passages in SPF ECE before duck inoculations. Since passage in ECE can produce an adaptation of the virus to the new host [37] and the selection of filamentous over the spherical forms of IAV [38], it is possible that this procedure might have affected the ability of the viruses to produce infection when approximately 10^2^ EID_50_ dose was used.

The results of MN assays suggested that heterosubtypic immunity occurred in the absence of detectable cross-neutralizing antibodies in serum against the viruses used for the second challenge (H4N6 or H6N2). Our findings are correlated with similar observations of HSI in mice where cross-neutralizing antibodies were not detected [39–41]. The lack of detection of neutralizing antibodies might be related to the existence of a detection threshold for the MN assay to detect cross-neutralizing antibodies in serum or its inability to identify antibodies that might be protective in vivo through antibody-dependent cellular cytotoxicity and complement activation [42]. Those findings should be taken into consideration when analyzing serological results of surveillance studies in mallards as lack of detection of antibodies against a specific subtype might not mean the absence of protective immunity against the IAV used for the assay in mallards. Also, further studies that assess the role cellular immunity in mallards and the observed cross-protection would enhance our understanding of HSI [43, 44].

In summary, we demonstrated that the required infectious dose to infect mallards with IAV can increase as a result of HSI induced by previous infections and that this effect is more pronounced when the HA of these viruses are more genetically related. Our findings suggest that the effect of the doses in the dynamics of IAV possibly influences the probability of transmission of viruses genetically related by the HA in mallards.

## Supporting information

S1 Table ELISA results after virus challenge. (XLSX)

S2 Table Individual microneutralization titers before and after heterosubtypic LPAIV inoculation against H4N6 or H6N2 antigens. (XLSX)

S3 Table Individual microneutralization titers before and after each virus challenge against the H3N8 antigen. (XLSX)
